# Automated severity level estimation of wheat rust using an EfficientNet-CBAM hybrid model

**DOI:** 10.3389/fpls.2025.1540642

**Published:** 2025-05-23

**Authors:** Sapna Nigam, Rajni Jain, Vaibhav Kumar Singh, Ashish Kumar Singh, Hari Krishna

**Affiliations:** ^1^ Division of Computer Applications, Indian Council of Agricultural Research (ICAR)-Indian Agricultural Statistics Research Institute, New Delhi, India; ^2^ Division of Technology and Sustainable Agriculture, Indian Council of Agricultural Research (ICAR)-National Institute of Agricultural Economics and Policy Research, New Delhi, India; ^3^ Division of Plant Pathology, Wheat Pathology Laboratory, Indian Council of Agricultural Research (ICAR)-Indian Agricultural Research Institute, New Delhi, India; ^4^ Computer Services Centre, Indian Institute of Technology Delhi, New Delhi, India; ^5^ Division of Genetics, Indian Council of Agricultural Research (ICAR)-Indian Agricultural Research Institute, New Delhi, India

**Keywords:** wheat rust, EfficientNet architecture, attention mechanism, disease severity estimation, transfer learning

## Abstract

Wheat rust is a severe fungal disease that significantly impacts wheat crops, resulting in substantial losses in quality and quantity, often exceeding 50%. Timely and early accurate estimation of disease severity in fields is critical for effective disease management. Early identification of Rust at low severity levels can facilitate prompt implementation of control measures, potentially saving crops. This paper introduces an automated wheat rust severity stage estimation model utilizing the EfficientNet architecture and attention mechanism. The convolutional Block Attention Module was integrated into EfficientNet-B0 in place of the SE module to enhance feature extraction by simultaneously considering channel and spatial information. The proposed hybrid approach aims to identify rust disease severity accurately. The model is trained on an image dataset comprising three major rust types—stripe, stem, leaf, and healthy plants captured under real-life field conditions. Each disease is categorized into four severity stages: healthy, low, medium, and high. Experimental results demonstrate that the proposed model achieves impressive performance, with a training accuracy of 99.51% and a testing accuracy of 96.68%. Moreover, comparative analysis against state-of-the-art CNN models highlights the superior performance of our approach. An Android application was also designed and developed to facilitate real-time classification of plant disease severity. This system incorporates a severity model optimized for enhanced classification accuracy and rapid recognition, ensuring efficient performance.

## 1 Introduction

Wheat ranks among the primary staple crops globally, with over half of its production dedicated to human consumption, livestock feed, and processing. However, wheat-producing nations face formidable challenges from plant diseases and pests, jeopardizing agricultural sustainability and profitability. Among these, rusts—comprising stripe, leaf, and stem Rust—stand out as particularly menacing fungal diseases, prevalent across almost all wheat-growing regions (Lu et al., 2017; Nigam and Jain, 2020). Left unchecked, rusts can mutate into virulent strains, leading to catastrophic crop failures. Conventional disease identification and severity assessment methods rely on manual visual inspection, which is fraught with inefficiencies, subjectivity, and labor intensiveness (Bock et al., 2010; Mohanty et al., 2016; Arnal Barbedo, 2019; Atila et al., 2021). Recent advances in computer vision, artificial intelligence (AI), and deep learning offer promising opportunities to automate disease detection and severity assessment through image analysis. In the existing literature, remarkable achievements in AI-based plant disease classification (LeCun et al., 2015; Fuentes et al., 2021; Arnal Barbedo, 2019; Chin et al., 2023; Pavithra et al., 2023; Dheeraj and Chand, 2022, 2024) underscore the potential of these technologies in integrated disease management.

However, while significant progress has been made in plant disease detection, much of the research has primarily focused on disease type classification, leaving a critical gap in the accurate quantification of disease severity. This gap limits experts’ ability to recommend optimal pesticide applications, compromising disease control efficacy and environmental sustainability. Thus, there is a growing demand for automated disease severity classification using AI-driven approaches (Esgario et al., 2020; Wspanialy and Moussa, 2020; Hu et al., 2021; Nigam et al., 2021). Disease severity, a crucial parameter for assessing the intensity of plant diseases, is traditionally quantified by comparing the diseased area of a plant part (such as leaves, fruits, or stems) to the total area of the affected part, based on standardized severity grading systems (Bock et al., 2022; Liu et al., 2020). For wheat stripe rust, severity evaluation is essential for effective monitoring, but it has primarily been carried out through visual observation, a method that requires experienced assessors and is both time-consuming and prone to errors (Jiang et al., 2022). Accurately estimating lesion areas according to severity standards is challenging, further complicating the process.

In contrast, disease incidence, which only requires determining whether a plant part is diseased or not, is easier to assess but does not provide a precise estimate of severity. The relationship between disease incidence and severity is influenced by factors such as lesion distribution, wheat plant resistance *to Puccinia striiformis (Pst*), and overall incidence levels (Chen et al., 2014), limiting the practical utility of incidence-based severity estimation methods. Recent machine learning advancements have provided some solutions for severity estimation. For instance, Wang et al. (2017) developed a model to predict disease severity at early, medium, and final stages, achieving notable accuracy. Similarly, Liang et al. (2019) introduced the PD^2^SE-Net model for horticultural crops, while Zhao et al. (2021) proposed SevNet, which uses ResNet and CBAM to classify tomato disease severity with impressive accuracies of 97.59% and 95.37%, respectively.

Despite these advances, research on cereal crop severity estimation remains limited due to the scarcity of image datasets. Notable exceptions include the BLSNet model for rice (Chen et al., 2021) and models for maize common rust severity prediction by Sibiya and Sumbwanyambe (2021) and Haque et al. (2022). Particularly underexplored is the classification of wheat yellow rust severity, with only one model—Yellow-Rust-Xception—proposed for differentiating yellow rust stages, achieving a modest 91% accuracy (Hayit et al., 2021). Also, Jiang et al., 2022 and Jiang et al., 2023 developed the machine learning models for severity assessment in wheat stripe rust. However, no deep learning-based model is developed in literature to estimate the severity of all three wheat rusts. This highlights the pressing need for further research and development to enhance the precision and reliability of disease severity assessments, particularly in wheat crops.

Moreover, challenges persist regarding the availability of public image databases, predominantly comprising lab-captured images rather than real-world field scenarios (Mi et al., 2020; Nigam et al., 2023). Hence, addressing these limitations is crucial for robust automated disease severity detection systems trained on datasets collected from natural field conditions.

Therefore, this paper focuses on the critical task of wheat disease severity classification, addressing the challenges associated with identifying and categorizing disease symptoms, understanding their impact on crop health, and exploring effective management strategies. The study emphasizes early detection of low-severity stages to mitigate crop loss and support sustainable agricultural practices. The main contributions of this research are summarized as follows:

- A comprehensive Wheat Disease Severity Dataset (WheatSev) was created, comprising 5,438 real-field images of wheat crops affected by stripe rust, leaf rust, and stem Rust across various growth stages.
- A convolutional block attention module (CBAM) was integrated into the EfficientNet B0 architecture to classify wheat disease severity into three levels: low, medium, and high. The CBAM-EfficientNet model demonstrated superior classification performance compared to several established architectures, including VGGNet19, ResNet152, MobileNetV2, DenseNet169, InceptionV3, and the original EfficientNet B0.
- The proposed model significantly improved classification performance in terms of accuracy, recall, precision, and F1 score by leveraging the combined strengths of EfficientNet B0 and CBAM layers. This approach effectively addressed technical challenges such as vanishing gradients and computational complexity while enhancing the robustness of the model.
- Robust data augmentation techniques were employed to increase data diversity, mitigating the risk of overfitting and ensuring the model’s reliability in classifying diverse real-world samples.
- The model’s efficiency was validated through extensive hyperparameter tuning, comparative analyses with state-of-the-art architectures, and Grad-CAM visualizations. Experimental results underscored the effectiveness of the CBAM-EfficientNet B0 model for accurate wheat disease severity estimation.

## 2 Materials and methods

This section delineates the tools, techniques, and procedures employed in the present study, including the acquisition of the image dataset and the proposed framework incorporating an attention module within the EfficientNet architecture.

### 2.1 Image dataset

Images depicting wheat rusts at various severity level stages were captured within the fields of ICAR-Indian Agricultural Research Institute, New Delhi, India, spanning three consecutive crop seasons. Image acquisition took place during sunny conditions, typically between 11:00 am to 1:00 pm, at ten-day intervals following the initial onset of disease symptoms. This timing ensured consistent leaf growth stages across all captured images.

**Figure 1:**
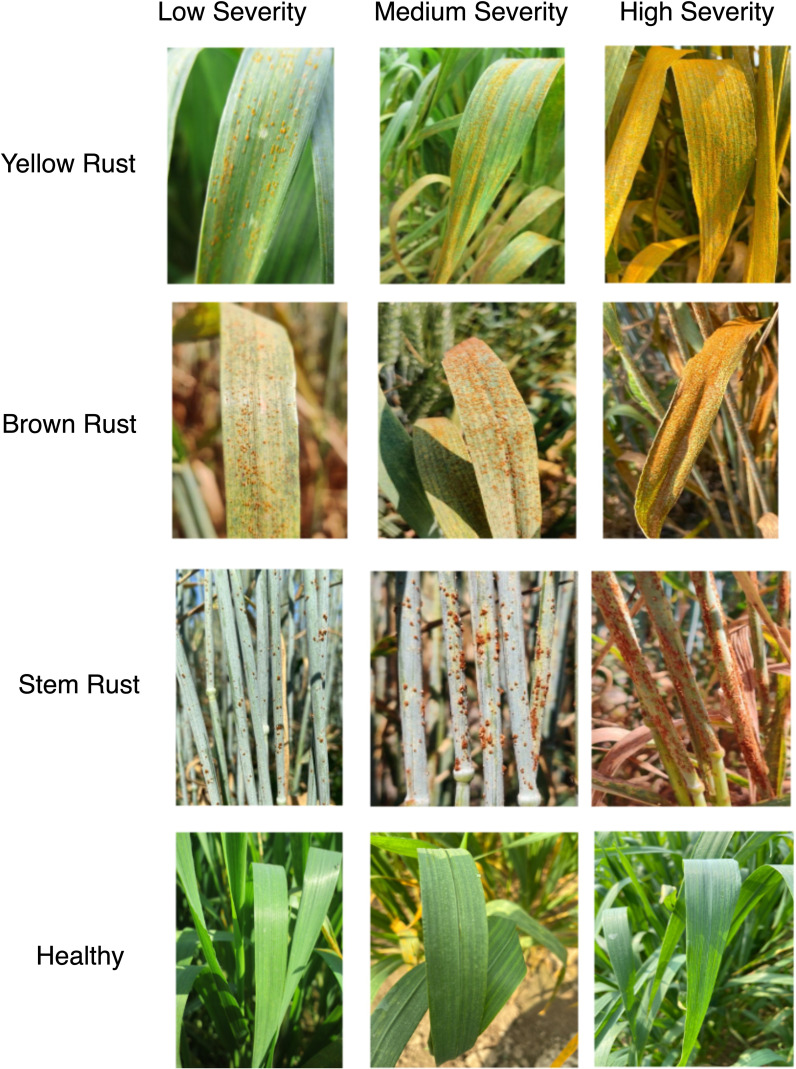
Wheat rusts images at severity levels: Low, Medium, High and Healthy.

A handheld mobile camera with a 20-megapixel resolution and a 25mm wide-angle lens was utilized for image acquisition. The deliberate use of mobile devices instead of professional cameras aimed to mirror the tools commonly employed by farmers in similar scenarios. The severity level estimation dataset encompasses images of three major wheat diseases, categorized into three severity levels by plant pathologists: low, medium, high and healthy (**Figure 1**). Images were captured by directing the camera lens toward regions of the leaves exhibiting disease symptoms at various growth stages. Subsequently, pathologists labeled the severity levels based on the percentage of disease symptoms present. Images categorized as low severity level exhibited severity symptoms ranging from 0 to 25 percent, those classified as medium severity level ranged from 25 to 50 percent, and high severity level images displayed more than 50 percent disease symptoms. The images with no disease symptoms were considered healthy as shown in **Figure 1**.

The primary emphasis of this study lies in low-level severity image classification to facilitate timely disease detection and mitigate crop loss. Initially, the severity stage estimation dataset comprised 5438 images distributed across ten classes, including three rust severity stages for each disease class and a class representing healthy leaves (refer to **Table 1**). To enhance classification performance and achieve balance among disease classes, the original dataset underwent augmentation, resulting in a total of 10252 images, with 1000 images allocated to each disease-infected class.

**Table 1:** Description of image dataset for severity stage estimation.

Disease	Class Name	# Original images	Severity level (%)	#Augmented images
No Disease	Healthy	1252	0	1252
Yellow Rust	YR_Low	216	0-25	1000
	YR_Medium	288	25-50	1000
	YR_High	501	>50	1000
Brown Rust	BR_Low	395	0-25	1000
	BR_Medium	595	25-50	1000
	BR_High	216	>50	1000
Stem Rust	SR_Low	780	0-25	1000
	SR_Medium	706	25-50	1000
	SR_High	489	>50	1000
Total no. of images		5438		10252

### 2.2 Data pre-processing and augmentation

Prior to model training, image pre-processing, and augmentation were performed to enhance model performance. Initially, duplicate, out-of-focus, noisy, or blurry photos were eliminated from the dataset to ensure data quality. Subsequently, the Augmentor Python package was utilized to augment the images by employing various techniques such as zooming, flipping, and rotating. This augmentation process aimed to diversify the dataset and enrich it with variations, thereby facilitating robust model training. Each class of severity stage images was augmented to contain 1000 images, ensuring balanced representation across classes (refer to **Table 1**). Additionally, the images were resized to 256 x 256 pixels to accommodate hardware constraints, optimize computational efficiency, and enhance the model’s generalization and performance. This pre-processing and augmentation pipeline laid the groundwork for effective model training on the augmented dataset.

### 2.3 Framework overview and structure

**Figure 2:**
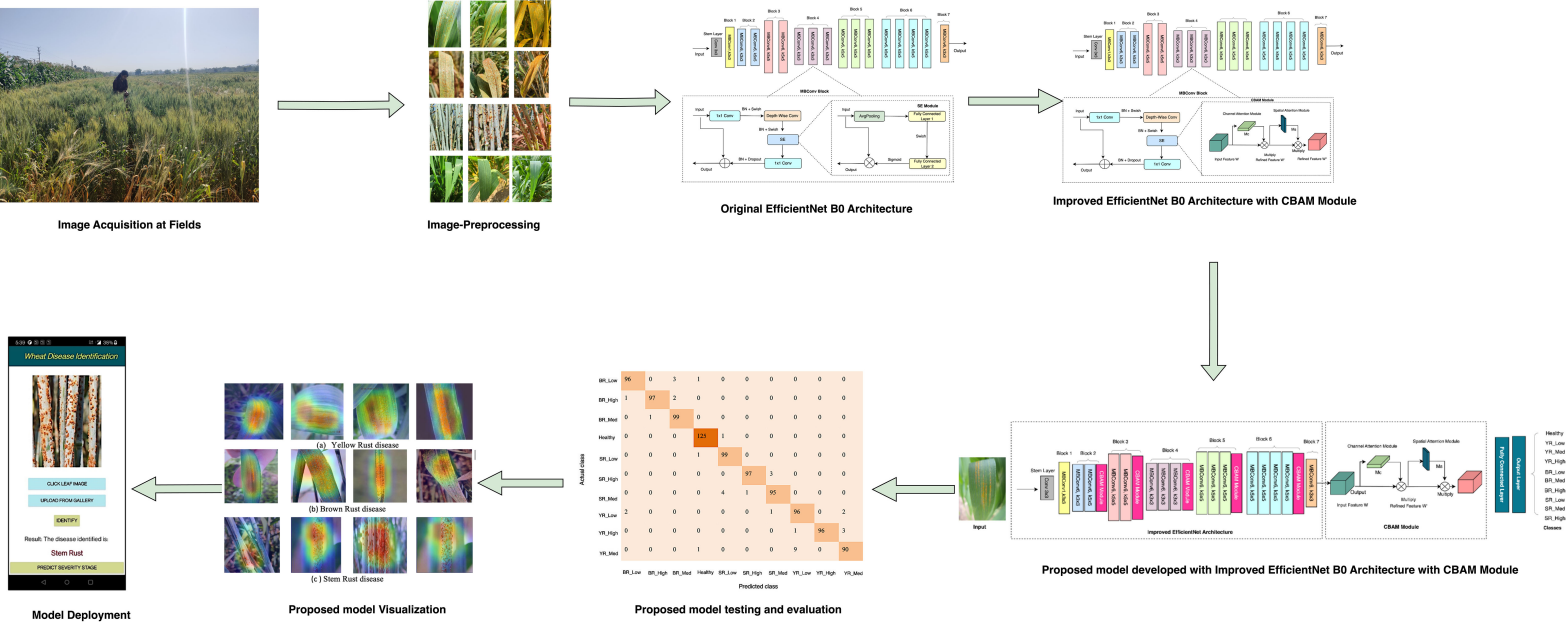
Overview of automated severity stage estimation framework.

The research methodology is visually depicted in **Figure 2**. Initially, images were captured from real-world wheat crop fields using mobile devices. Domain experts labeled each image with the corresponding type of wheat rust and its severity level, organizing them into distinct folders. Subsequently, image processing techniques, including resizing, filtering, and noise reduction, were applied to refine the raw images. Augmentation techniques, such as random rotation, translation, flipping, and zooming, were employed to diversify the image dataset and validate the models before experimentation. Two datasets were created: the original dataset containing 5438 images and an augmented dataset comprising 10252 images, both segregated into train, test, and validation sets in an 80:10:10 ratio for experimentation purposes. Initially, the performance of the fine-tuned EfficientNet B0 model was evaluated on both datasets. Subsequently, to enhance the model’s performance, the proposed model was developed by integrating the CBAM module (Tan and Le, 2020) into the fine-tuned EfficientNet B0 model. The attention mechanism’s channel and spatial modules focus on key disease symptoms, aiding in determining the severity level of wheat rust. **Figure 2** illustrates the flowchart of the wheat disease identification and severity stage estimation framework, with subsequent sections elaborating on each phase of the framework.

### 2.4 Architectural overview of EfficientNet and attention mechanism integration

**Figure 3:**
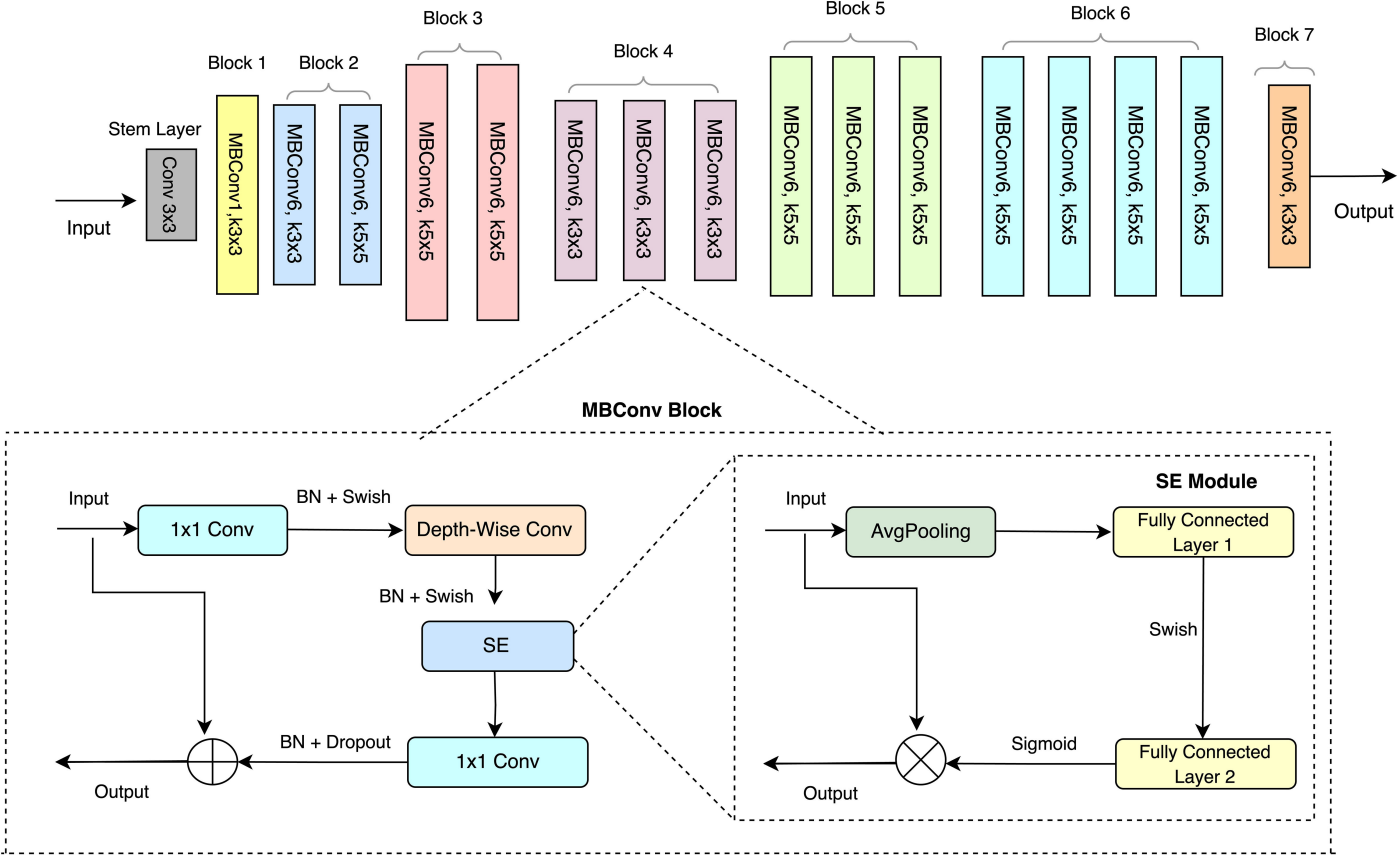
Baseline architecture of EfficientNet B0.

The EfficientNet B0 serves as the foundational model within the EfficientNet family, encompassing a total of eight variants (B0-B7) (**Figure 3**). EfficientNet B0 architecture achieves high accuracy and computational efficiency through a compound scaling approach as described by Atila et al. (2021). The EfficientNet architecture employs the Mobile Inverted Bottleneck Convolution (MBConv) as its primary building block, introduced by Sandler et al. (2018) and illustrated in **Figure 3**. The MBConv block comprises three components: a 1 × 1 convolution (1 × 1 Conv), Depth-wise convolution (Depth-wise Conv), and a Squeeze-and-Excitation (SE) module. Initially, the output of the preceding layer is passed through the MBConv block, where the number of channels is expanded using a 1 × 1 Conv. Subsequently, a 3 × 3 Depth-wise Conv reduces the number of parameters, followed by channel pruning that compresses the channel count through another 1 × 1 Conv. A residual connection is then introduced between the input and output of the projection layer to enhance feature representation. The SE module, as shown in **Figure 3**, incorporates two key operations: squeeze and excitation. The squeeze operation is performed using global average pooling (AvgPooling), while the excitation operation involves two fully connected layers activated sequentially with a Swish activation and a Sigmoid activation function. This design facilitates efficient parameter utilization while maintaining high performance. However, the SE module focuses on channel-wise feature recalibration by emphasizing informative channel characteristics while suppressing less relevant ones. However, this approach primarily addresses channel-specific information and overlooks spatial context, which is critical for visual recognition tasks such as severity estimation. This limitation negatively affected the model’s classification accuracy for severity estimation.

**Figure 4:**
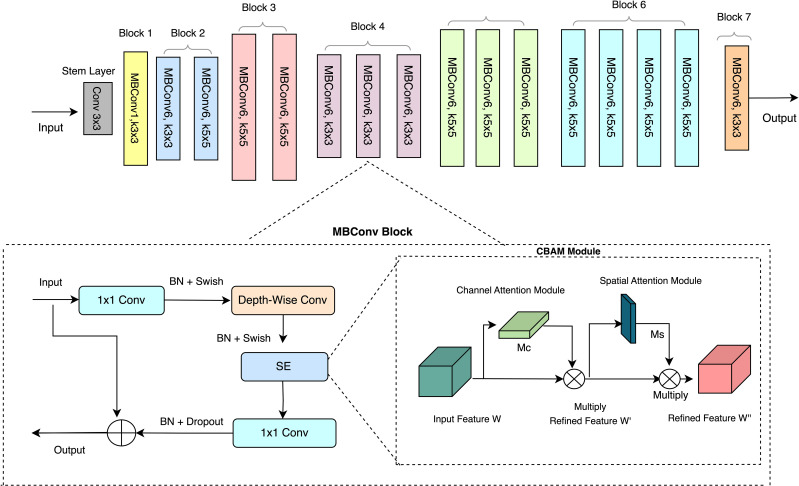
Improved EfficientNet B0 architecture: SE module replaced with CBAM module.

To address this, the (CBAM) was integrated into EfficientNet-B0 in place of the SE module to enhance feature extraction by simultaneously considering both channel and spatial information. The modified network, referred to as EfficientNet-CBAM, is illustrated in **Figure 4**. Key modifications include the replacement of the SE module in each MBConv layer with a CBAM module, allowing the network to retain vital spatial information alongside channel-specific features, particularly for identifying disease severity symptoms. Additionally, a CBAM module was introduced after the final convolutional layer, refining the extracted features and improving the network’s classification performance. The final convolutional layer of EfficientNet B0 produces feature maps, which serve as input for the Convolutional Block Attention Mechanism (CBAM) module (see **Figure 4**).

**Figure 5:**
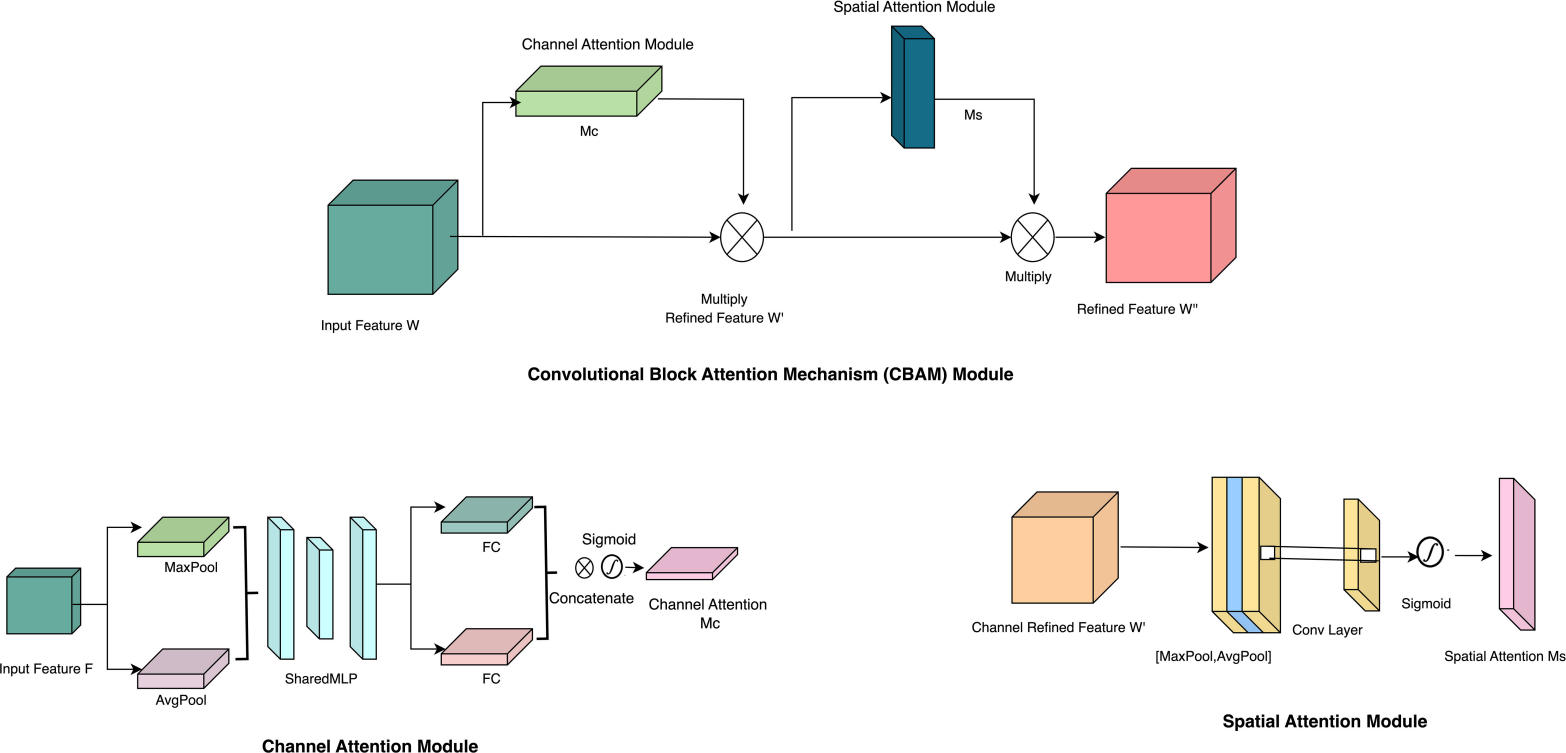
Overall architecture of CBAM module.


Attention mechanisms, extensively utilized in research, augment feature extraction and boost model performance in image classification tasks (Woo et al., 2018; Wang et al., 2017). Our architectural design incorporates a convolutional block attention module with two key components: the Channel Attention Module (CAM) and the Spatial Attention Module (SAM) (refer to **Figures 5**). These two modules work together to improve feature extraction and representation within the generated feature maps (Woo et al., 2018). The input feature map W, representing the wheat rust-infected leaf image, undergoes processing within the CAM, producing the channel attention feature map Mc. This map highlights essential image information, which is then used to generate the refined feature map W’. Element-wise multiplication between M_c_ and W yields the improved feature map W’. Subsequently, W’ is subjected to processing within the Spatial Attention Module, generating the spatial feature map M_s_. This map selectively emphasizes significant image areas. The enhanced feature map W’ is subsequently combined with the spatial feature map M_s_. This multiplication yields the ultimate feature map W’’, which encapsulates the representation of the wheat rust image. The Convolutional Block Attention Mechanism operates through the following Equations 1, 2:

(1) W'=Mc(W)⊗W

(2) W”=Ms(W')⊗W'

The CBAM module’s channel attention mechanism utilizes pooling operations to compress the feature map W, focusing solely on essential symptom regions within the image while disregarding extraneous information or features. Conversely, the spatial attention mechanism identifies significant feature locations post-CAM processing. This process involves spatial dimension compression of the feature maps W’ and the generation of the spatial attention feature M_s_ utilizing the sigmoid activation function. It highlights critical features within specific image area, enhancing intermediate features.

### 2.5 Proposed severity estimation framework

**Figure 6:**
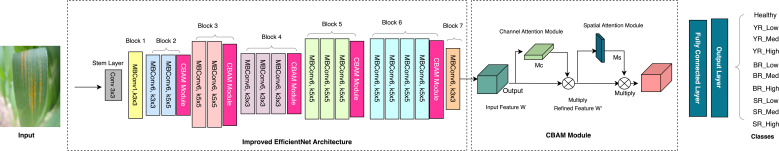
Overall proposed model framework for Wheat disease severity estimation.

The proposed methodology employs transfer learning, where a novel model aimed at disease severity stage identification is trained utilizing a pre-trained model, EfficientNet B0, as the foundation for learning. While retaining the initial layers of the EfficientNet B0 model, the final layer is replaced with new layers. These newly introduced layers are subsequently fine-tuned to classify infected leaves into ten distinct classes using, ‘WheatSev’ dataset developed by us, as per the methodology given by Too et al. (2019). Thus, the WheatSevNet model is designed to accurately classify the severity stages of wheat rust infections by enhancing feature extraction and representation. It builds upon EfficientNet-B0, a widely used deep learning architecture known for its computational efficiency and high performance. However, EfficientNet-B0’s Squeeze-and-Excitation (SE) module, while effective for channel-wise recalibration, lacks spatial feature extraction capabilities. To overcome this limitation, WheatSevNet integrates the Convolutional Block Attention Module (CBAM) in place of the SE module within each MBConv block of EfficientNet-B0 as depicted in **Figure 6**. The CBAM module consists of two components: the Channel Attention Module (CAM), which selectively enhances significant channels using global average pooling, max pooling, a multi-layer perceptron (MLP), and a sigmoid activation function, and the Spatial Attention Module (SAM), which refines feature extraction by applying average and max pooling across the channel axis, followed by a convolutional layer and a sigmoid activation function. In WheatSevNet, each MBConv block of EfficientNet-B0 is modified by replacing the SE module with CBAM, ensuring both spatial and channel-wise attention are effectively incorporated. Additionally, a final CBAM layer is introduced after the last convolutional layer to further refine feature maps before classification. The model leverages transfer learning and fine-tuning, utilizing pre-trained EfficientNet-B0 weights with the initial layers frozen while optimizing the later layers for severity classification. Further modification involves the addition of a normalization layers, fully connected (FC) layer, a dropout, and a convolutional layer, as depicted in **Figure 6** for detecting and classifying different severity stages of wheat rust. By integrating CBAM at multiple levels, WheatSevNet achieves enhanced feature extraction, capturing both disease-specific spatial structures and critical channel-wise characteristics, thereby improving classification performance over traditional EfficientNet-based approaches.

Additionally, **Table 2** presents the hyperparameters employed for the disease severity estimation models. Owing to low stopping, the number of epochs ranged from 27 to 50, with a fixed learning rate of 0.001. To mitigate this risk, the authors have incorporated several measures to prevent overfitting. These include data augmentation, which helps in artificially increasing the dataset size and providing more diverse training examples. The augmentation process involved applying random transformations such as rotation, flipping, scaling, and color adjustments to create new variations of the existing images. This technique aimed to simulate real-world variations in the data, which helps prevent the model from overfitting to the specifics of the training set. Furthermore, class balancing was ensured by augmenting each class of severity stage images to contain a total of 1000 images per class, ensuring that all classes were equally represented in the dataset. This balanced representation prevents the model from being biased towards any particular class, enhancing its ability to generalize across different categories. Further, a dropout rate of 0.20 was implemented during the training process. Dropout works by randomly disabling 20% of the neurons in each layer during training, which helps prevent the model from becoming overly reliant on specific features and encourages it to learn more generalized patterns. Additionally, L2 regularization has been applied to control the complexity of the model and prevent it from overfitting to the training data. In addition to these measures, early stopping with patience as 3 was incorporated as an extra safeguard against overfitting. This technique monitors the validation loss during training, and if no improvement is seen for a specified number of epochs (the patience parameter), the training process is halted early. This prevents the model from continuing to learn noise and overfitting to the training data. The patience parameter was set to allow the model to train for several epochs without improvement before stopping, ensuring that it had enough time to converge but also preventing unnecessary overfitting. All these hyperparameters for the severity estimation models are reported in **Table 2**. Categorical cross-entropy served as the loss function during model training, while the batch size for experimentation was fixed at 32. The subsequent subsection will address the third objective of the research study, focusing on elucidating the validation of the developed models and their integration into mobile applications.

**Table 2:** Hyperparameters set for wheat disease severity model.

Epochs	Batch size	Optimizer	Learning rate	Momentum	Loss function	Dropout
27-50	32	Adamax	0.001	0.99	Categorical cross-entropy	0.20

### 2.6 Evaluating model performance and efficacy

During the experiment, various pre-trained classical deep-learning models were compared to the proposed model. These models included VGGNet (Simonyan and Zisserman, 2014), ResNet152 (He et al., 2016), InceptionV3 (Szegedy et al., 2016), MobileNetV2 (Sandler et al., 2018), and DenseNet121 (Huang et al., 2017). These models underwent parameter resetting before training, followed by modifications to the bottom layers of the pre-trained networks. The bottom layer was substituted with a new SoftMax and output layers containing ten severity stage classes from the datasets.

### 2.7 Experimental implementation

The experimentation was conducted on a robust DGX server featuring GPU capabilities, with computations executed using the Keras and TensorFlow frameworks. The system has Ubuntu as the operating system, supported by an Intel^®^ Xeon^®^ CPU. All computationally intensive tasks were handled by the NVIDIA Tesla V100-SXM2 GPU, boasting ample memory resources of 528 GB (refer to **Table 3**).

**Table 3:** Experimental setup.

Operating system	GPU	Memory	Frameworks	Programming language	System
Ubuntu	NVIDIA Tesla V100-SXM2	528 GB	Keras and TensorFlow	Python	Intel^®^ Xeon^®^

### 2.8 Evaluation metrics

The accuracy of classification predictions in machine learning experiments is assessed through confusion matrices. It is used to analyze the correspondence between the predicted and actual prediction scores for individual classes of a classification model. Other metrics such as precision, accuracy, F1 score, and recall are also used to assess the performance of our model. Precision describes the ratio of true positives to all positive predictions, while accuracy refers to the proportion of correctly identified predictions relative to the total number of the predictions. On the other hand, Recall measures the ratio of true positive cases to all positive predictions (Hossin and Sulaiman, 2015). The F1 score is further calculated using the harmonic mean of precision and recall, providing a balanced evaluation of the model performance (Equations 3–6).


(3) $$\text{Precision}=\;\frac{\text{TP}}{\text{TP}+\text{FP}}$$

(4) $$\text{Accuracy}=\;\frac{\text{TP}+\text{TN}}{\text{TP}+\text{TN}+\text{FP}+\text{FN}}$$

(5) $$\text{Recall}=\;\frac{\text{TP}}{\text{TP}+\text{FN}}$$

(6) $$\text{F}1=\;\frac{2\text{TP}}{2\text{TP}+\text{FP}+\text{FN}}$$

The “true positive” (TP) represents the count of images accurately detected within each severity stage class. Conversely, “true negative” (TN) represents the overall number of images correctly identified across all severity stages, excluding the specific severity stage to which they belong. “False negative” refers to the count of images wrongly classified within each relevant severity class, while “false positive” (FP) indicates the number of images incorrectly classified as belonging to different severity stage classifications. Finally, the predictive performance of the proposed model is summarized by the F1 score.

## 3 Results and discussion

The experiment aimed to estimate the severity stages for all three wheat rusts utilizing the image dataset. The wheat disease severity estimation model was crafted using the EfficientNet architecture as the foundational model, augmented with the Convolutional Block Attention Mechanism (CBAM) integrated at the network’s base. Performance evaluation of the disease severity estimation model was conducted, posing it against state-of-the-art CNN models and a simple fine-tuned EfficientNet B0 model, as outlined in **Table 4**. Results demonstrate that the proposed severity model achieved the highest test accuracy, reaching 93.88% and 96.68% on both the non-augmented and augmented datasets, respectively. Upon comparing the experimental results of EfficientNet B0 and EfficientNet B0-CBAM, as presented in **Table 4**, a notable enhancement in disease severity identification was observed upon the integration of an attention mechanism into the model.

**Table 4:** Performance comparison of State-of-the-art CNN models with proposed severity estimation model.

Model parameters			Accuracies on non- augmented dataset (%)			Accuracies on augmented dataset (%)		
Model	Image size	Ep	Training	Validation	Testing	Training	Validation	Testing
VGGNet19	224*224	50	85.14	84.78	83.27	86.73	85.09	85.12
ResNet152	224*224	50	87.72	86.03	86.91	88.14	87.65	87.08
MobileNetV2	224*224	50	86.90	85.87	84.78	87.05	86.31	84.93
DenseNet169	224*224	50	90.13	90.15	89.97	91.44	90.7	90.56
InceptionV3	299*299	50	91.82	90.50	90.22	94.62	92.05	91.84
EfficientNet B0	224*224	25	96.33	92.68	91.56	98.54	93.43	92.19
Proposed model	224*224	27	98.67	94.83	93.88	99.51	95.97	96.68

**Figure 7:**
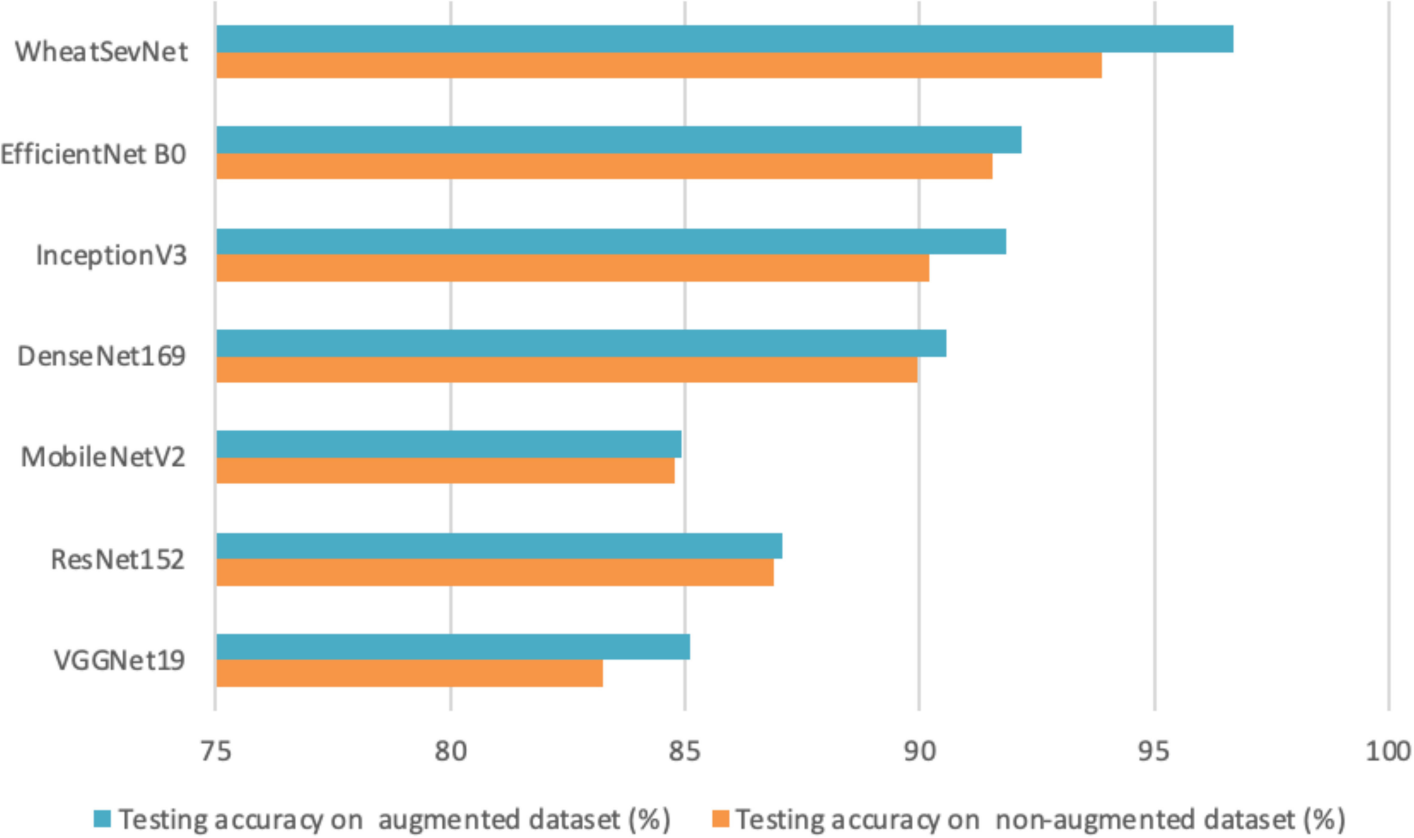
Comparison of proposed model testing accuracies with various CNN models.

In the absence of an attention mechanism in EfficientNetB0, the overall testing accuracy on the WheatSev dataset was recorded at 92.19%. However, upon integrating the CBAM module into EfficientNetB0, the overall testing accuracy markedly increased to 96.68%, as illustrated in **Figure 7**. Analysis of the disease severity stage classification results revealed that the performance enhancement observed in EfficientNetB0, when augmented with the attention mechanism, could be attributed to the spatial attention module of the CBAM module, which adeptly locates key information with greater accuracy. Furthermore, the channel attention module of CBAM exhibits the ability to amplify important features while suppressing irrelevant ones, thereby yielding a more refined feature representation. Consequently, it can be inferred that the incorporation of the CBAM module into the base model effectively contributes to improving the model’s performance in identifying the severity level of wheat rust diseases.

### 3.1 Confusion matrix and various performance metrics

**Figure 8:**
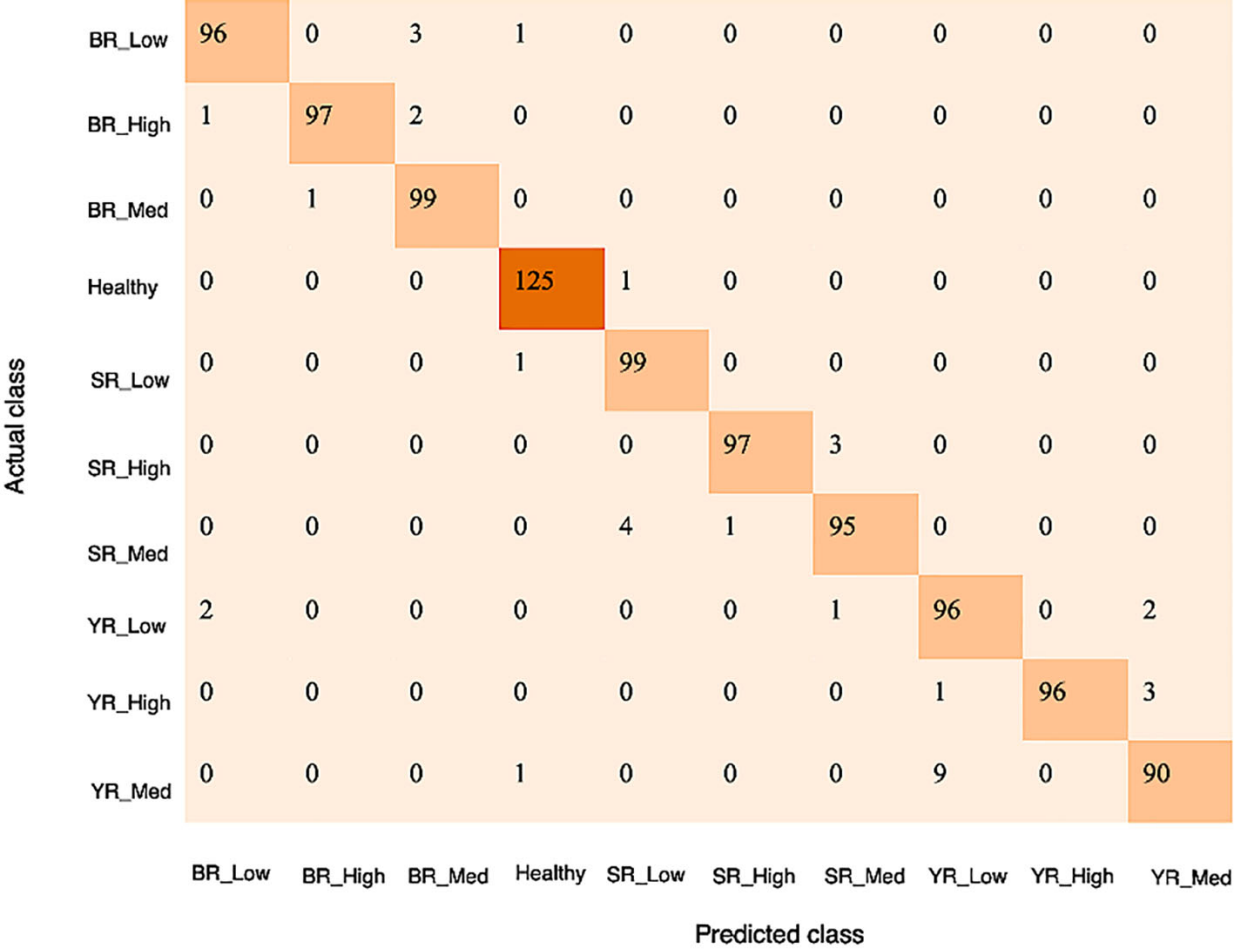
Confusion matrix for proposed disease severity estimation model.

**Figure 8** illustrates the confusion matrix of the severity estimation model, depicting ten classes based on actual and predicted labels from augmented datasets. The numbers along the diagonal signify accurately identified images, while those outside the diagonal indicate instances of misclassification (Ting, 2017). Specifically, among the four low severity images of brown Rust, two were erroneously identified as medium-stage severity, and one as healthy (**Figure 8**). Similarly, misclassifications occur in other severity stages of brown Rust, with images of high and medium levels misclassified as medium and low, respectively. Furthermore, low-severity images of brown Rust were mistakenly classified as medium severity and healthy. Additionally, misclassifications were observed in stem rust and yellow rust severity stages.

Upon analyzing the confusion matrix of EfficientNetB0 embedded with CBAM, it was noted that Rust spread on the upper surface of the leaf leads to significant confusion between the low and medium severity stages of the disease. Referencing **Table 5**, the classification report derived from the confusion matrices includes F1, precision, and recall metrics for the proposed disease severity estimation model. A model is deemed appropriate if its F1 score approaches one. After evaluating these performance metrics, the following findings emerge: On the augmented dataset, the average precision, recall, and F1 score for identifying severity stages in brown rust and stem rust is 97%, whereas, for yellow Rust, the average score for precision, recall, and F1 measure is 95%.

**Table 5:** Performance metrics for severity estimation model on both datasets.

Classes	Augmented dataset			Non-augmented dataset		
	Precision	Recall	F1	Precision	Recall	F1
Healthy	0.98	0.99	0.99	0.96	0.99	0.97
BR_Low	0.97	0.96	0.96	0.9	0.84	0.87
BR_Medium	0.95	0.98	0.97	0.8	0.75	0.82
BR_High	0.99	0.98	0.98	0.88	0.95	0.91
SR_Low	0.95	0.99	0.97	0.95	0.94	0.94
SR_Medium	0.97	0.95	0.96	0.9	0.91	0.91
SR_High	0.99	0.97	0.98	0.93	0.94	0.93
YR_Low	0.91	0.96	0.94	0.8	0.81	0.8
YR_Medium	0.95	0.91	0.93	0.78	0.75	0.77
YR_High	1	0.97	0.98	0.95	0.92	0.93

Shades of red color for higher, yellow for moderate, and green for lower values of performance parameters.

**Table 5** shows that augmented datasets yield superior results compared to non-augmented datasets across all diseases and their categories. However, for stem rust disease, all performance parameters in non-augmented datasets surpass 90% across all stages, possibly due to easily identifiable features of stem rusts. Conversely, for brown and yellow Rust, the performance of non-augmented datasets is notably inferior to augmented datasets. Another noteworthy finding pertains to the higher classification accuracy of healthy leaves, which can be attributed to (i) the more significant number of images (1252) and (ii) the absence of any classes for healthy leaves. Although the accuracy improves as the disease stage matures, even in low stages, precision exceeds 90% for all types of rusts. For the augmented dataset, precision reaches 97% for brown Rust and 91% for yellow Rust.

### 3.2 Model accuracy and loss curves

**Figure 9:**
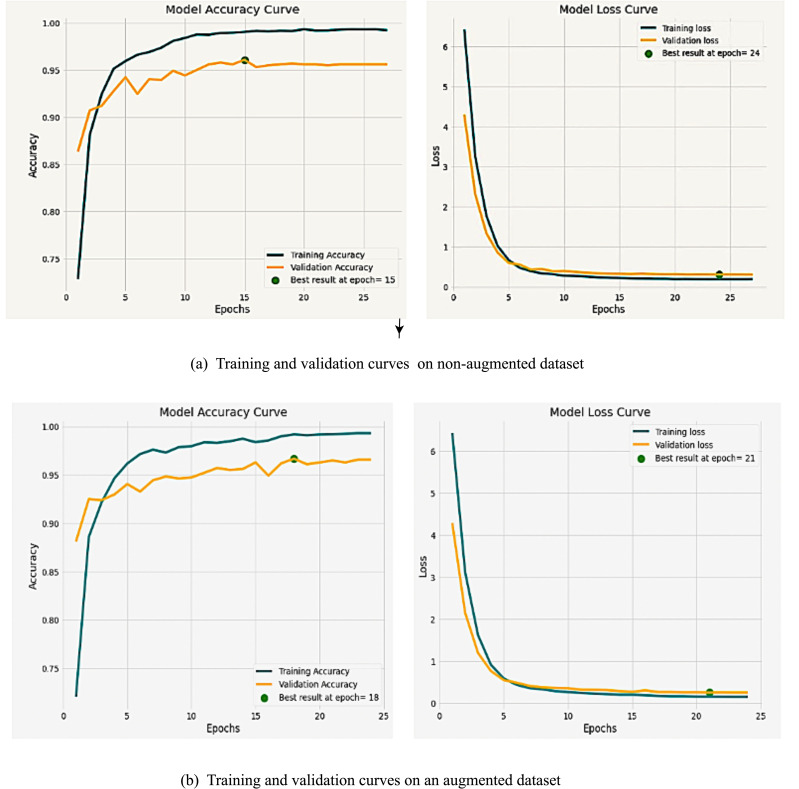
Training and validation curves for severity estimation model on **(a)** non-augmented dataset **(b)** augmented dataset.

**Figure 9** depicts the training and validation curves for the wheat severity estimation model, offering insights into the learning process. Notably, the accuracy curves indicate that our proposed severity model serves as a commendable and efficient fit model (Hossin and Sulaiman, 2015). The findings of the disease severity model underscore its efficacy in automatically identifying wheat severity stages based on images.

In conclusion, the proposed models prove useful for identifying diseases at a low severity level, as evidenced by the accurate identification of most images in the low stages of Rust. This early severity assessment holds promise for crop preservation and can significantly minimize crop loss.

### 3.3 Visualization of disease symptoms model interpretability

**Figure 10:**
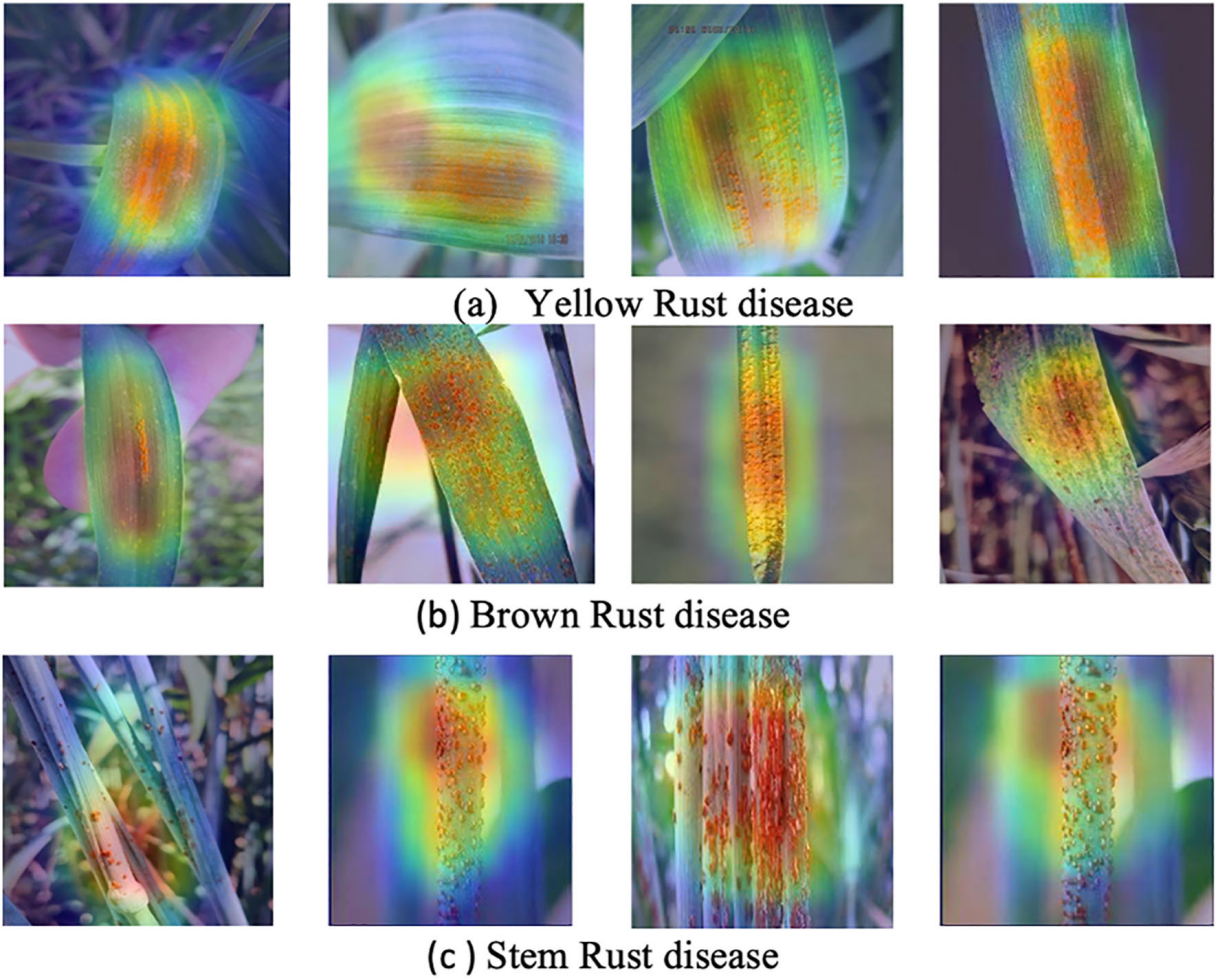
GradCAM visualization of the **(a)** Yellow rust **(b)** Brown rust **(c)** Stem rust diseases at low severity level.

The interpretability of the proposed model is carried out using the GradCAM (Selvaraju et al., 2020). **Figure 10** illustrates that the proposed severity estimation model focuses explicitly on the features and the symptoms that play an important role in identifying the severity level of the type of the wheat rusts. The activation maps shown in the figure facilitate specific regions in the input test images necessary for estimating the severity of the disease. Thus, we aimed to illustrate how the model is directing its attention towards the areas where the symptoms are most noticeable in order to identify the disease at a low stage of severity. The attention mechanism has been found to improve the model’s ability to identify the appropriate symptoms in the correct location accurately.

### 3.4 Development of an Android based mobile application for wheat disease severity estimation

**Figure 11:**
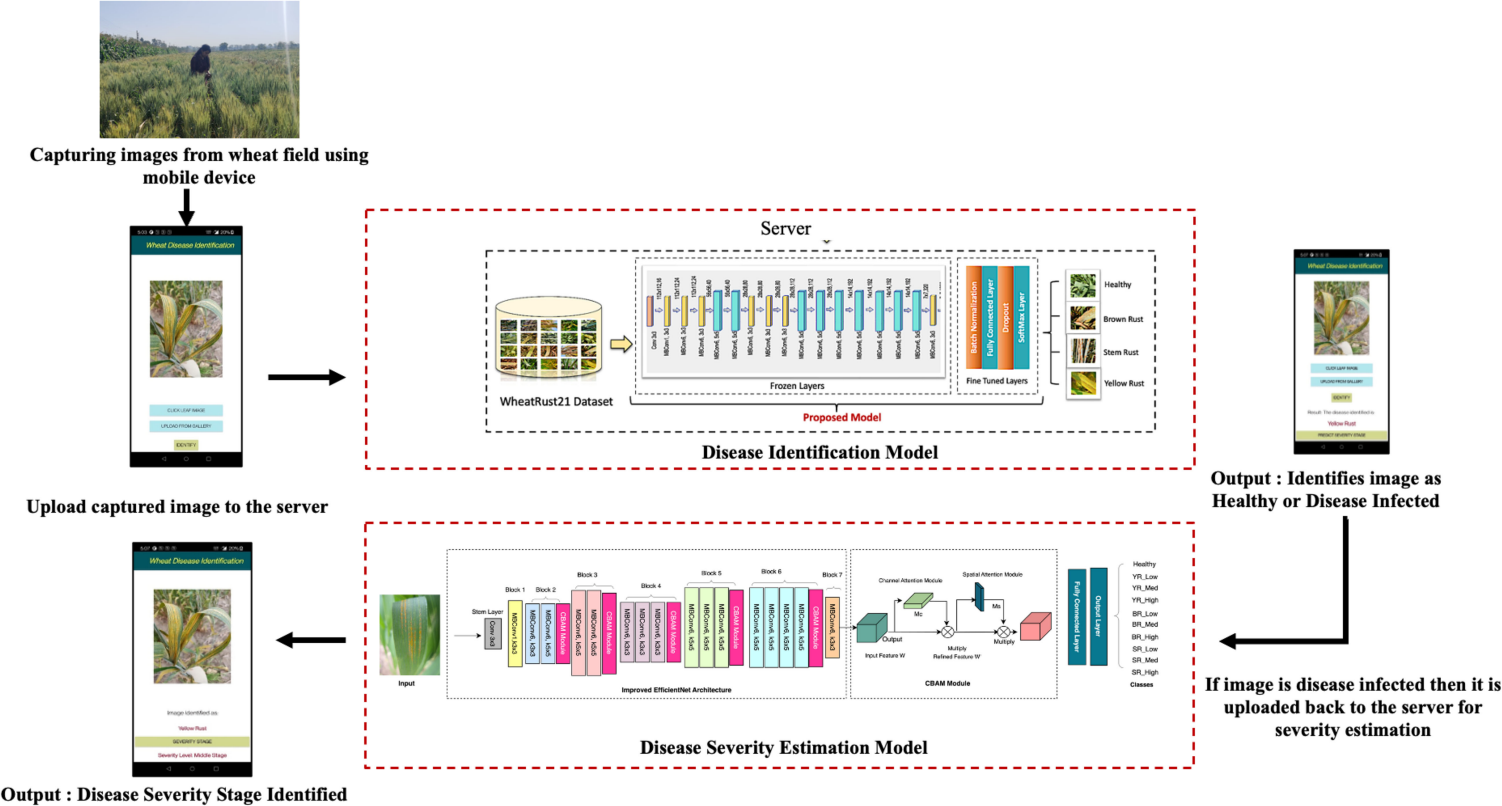
Overall flow of disease severity estimation through mobile application.

In this study, we also developed an Android-based mobile application as a practical tool for the automatic identification of wheat diseases and their corresponding severity stages in agricultural fields. The proposed model was integrated into the application’s backend to facilitate this functionality. The mobile application allows users to capture real-time images from agricultural fields or select images stored in their mobile device gallery. These images are subsequently uploaded to a server for analysis. The analytical process begins with the application determining whether the uploaded image depicts a healthy or diseased wheat plant. For images identified as healthy, the result is directly displayed as “healthy”. Conversely, if the image is diagnosed as diseased, the application proceeds to identify the specific type of disease. Following disease identification, the application further evaluates the image to estimate the severity stage of the detected disease. **Figure 11** provides a detailed illustration of the application’s process flow, from image acquisition to disease identification and severity stage estimation.

**Figure 12:**
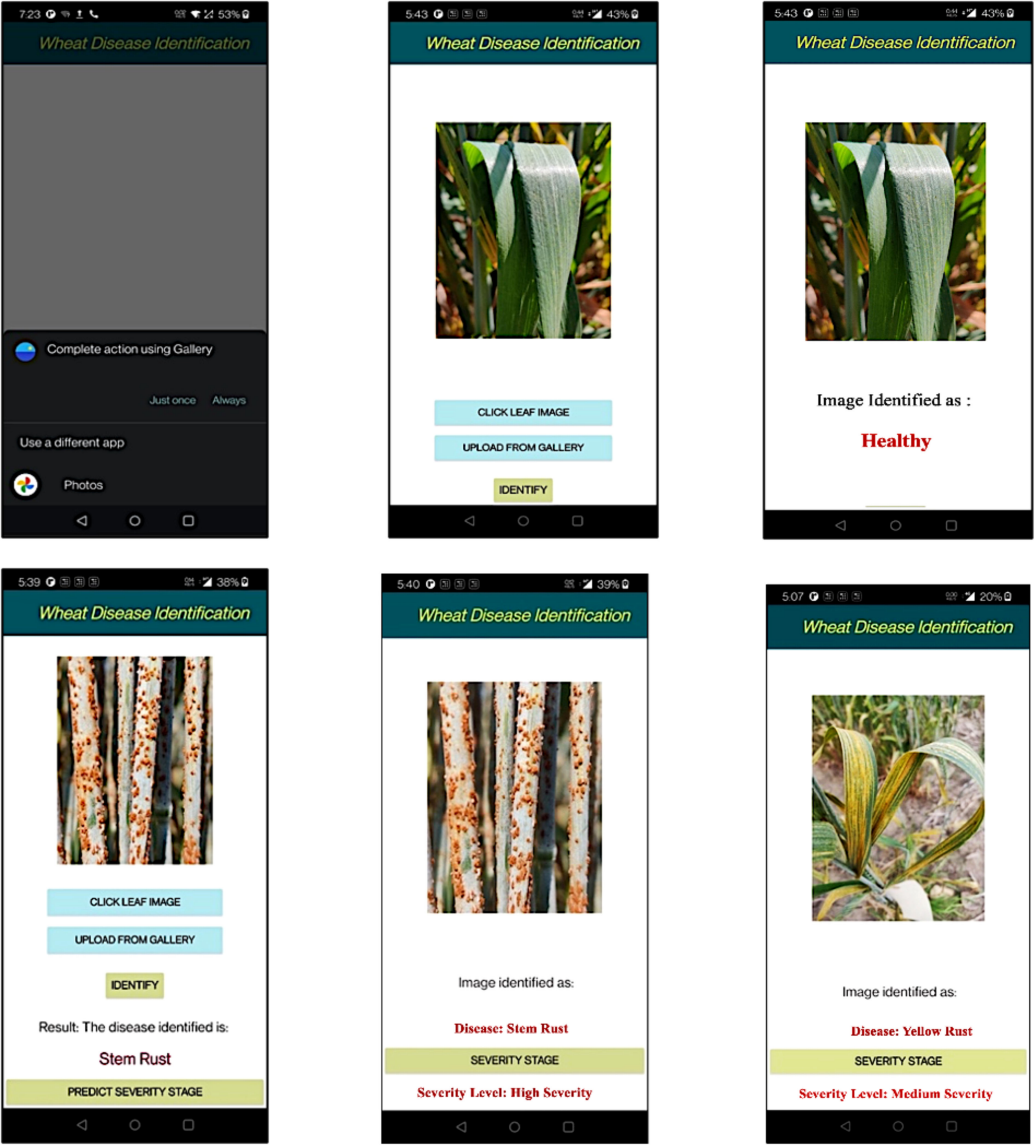
Screenshots of the developed mobile application for severity estimation.

The developed mobile application for wheat disease severity estimation follows a streamlined workflow **Figure 12**. Upon launching, a splash screen introduces the app, followed by an interface that allows users to capture or upload a wheat leaf image. Once an image is uploaded, the “Identify” button determines if the leaf is healthy or diseased. For healthy images, the app displays a message indicating no further action is required. If a disease is detected, the application identifies the disease type and provides an option to predict its severity stage. The final screen presents the identified disease along with its severity stage, providing a complete diagnostic result for the uploaded image.

### 3.5 Comparison with existing studies in the literature

The **Table 6** offers a comprehensive overview and comparative assessment of various models utilized for disease classification across different crops, alongside their respective training or testing accuracies. Our proposed model, specifically for diagnosing three major rusts in wheat crops, achieved a commendable testing accuracy of 96.68%. When compared with existing literature, our model emerges as a strong contender, demonstrating competitive performance. Notably, existing models developed for crops such as apple, tomato, coffee, cucumber, and grape achieved accuracies ranging from 90.4% to 97.75%, albeit focusing on single disease classes. Importantly, prior attempts at wheat severity estimation encompassing three diseases and their severity levels were scarce. Despite this, our model’s accuracy not only matches but also exceeds the reported accuracies in the literature, underscoring its efficacy in wheat rust severity estimation.

**Table 6:** Summarization of the previous studies for image-based disease severity estimation.

References	Crops/diseases/classes	No. of diseases	Algorithm/ Architecture	Training/Testing accuracy (%)	Dataset with number of images
Wang et al. (2017)	Apple (Leaf black rot)	1	VGG16 model	90.4	PlantVillage (1986)
Liang et al. (2019)	Multiple fruit crops (healthy, general, serious)	–	ResNet & ShuffleNet	91	PlantVillage
Prabhakar et al. (2020)	Tomato (early blight) (mild, moderate, severe)	1	ResNet101	94.6	PlantVillage
Verma et al. (2020)	Tomato (Late blight)	1	AlexNet	93.4	PlantVillage
Esgario et al. (2020)	Coffee (Leaf biotic stress)	1	ResNet50	95.24	Own (2293)
Wang et al. (2021)	Cucumber (downy and powdery mildew)	2	DeepLabV3	92.85	Own (1000)
Zhao et al. (2021)	Tomato (fungal diseases)	–	SENet & CBAM	95.37	PlantVillage
Liu et al. (2022)	Apple (Alternaria leaf blotch)	1	DeepLabV3, UNet	96.41	Own (5382)
Ji and Wu et al. (2022)	Grape (black measles)	1	ResNet & Fuzzy logic	97.75	PlantVillage
Li et al. (2023)	Wheat (yellow Rust)	1	GhostNetV2	95.44	Public
Hu et al. (2023)	Tea (leaf blight)	1	GBM	–	Own (300)
WheatSevNet (Proposed)	Wheat (three major rusts)	3	EfficientNet & CBAM	96.68	Own (10000)

In this study, our primary contributions are twofold: Firstly, we curated a robust dataset for wheat disease classification, encompassing the estimation of severity categories. The non-augmented dataset comprises 5438 images, while the augmented dataset boasts a total of 10252 images. This dataset lays a strong foundation for future research in this domain. Secondly, we introduced WheatSevNet, an algorithm capable of identifying various wheat disease categories and assessing their severity levels. Despite the challenges posed by multi-disease classes and multi-severity levels, our enhanced model achieved an impressive accuracy rate of over 96%. This performance is comparable even to other algorithms designed for single disease identification. We could not compare with wheat severity estimation models for three diseases as no such published attempt is available to the best of our knowledge. The success of our approach not only addresses an immediate need in agricultural research and opens up promising avenues for future investigations in this field. We hope our study will inspire further exploration and innovation in automated plant disease diagnosis and severity estimation.

## 4 Conclusion

The major fungal diseases in a wheat crop significantly impact crop quality and quantity, leading to substantial agricultural yield losses. In our study, we aim to diagnose major wheat fungal diseases and its corresponding severity level, utilizing a model based on EfficientNet architecture and enhanced with a Convolutional Block Attention Mechanism. The proposed model demonstrates exceptional effectiveness, boasting a training accuracy of 99.51% and a testing accuracy of 96.68%. In comparative analyses, our model surpasses state-of-the-art CNN models and a fine-tuned EfficientNet B0 model, highlighting its superior performance in severity estimation. To ensure the robustness of our approach across various disease categories, we conducted experiments using images from real-life field conditions, encompassing three major types of wheat rusts: yellow, brown, and black. Notably, our model’s ability to classify severity stages into medium and high stages provides precise information, facilitating timely intervention. The integration of the CBAM module significantly enhances the model’s performance, boosting the testing accuracy from 93.21% to an impressive 96.68% on the WheatSev dataset. This improvement is largely attributed to the attention module within CBAM, which adeptly identifies critical information and enhances the representation of features. Furthermore, the channel attention module demonstrates its effectiveness in amplifying features while suppressing ones, thereby contributing to a more precise and accurate identification of the severity level of wheat rust disease. The results validate that the inclusion of the CBAM module substantially improves the efficiency of the model in detecting and assessing the severity of wheat rust disease.

## Data Availability

The raw data supporting the conclusions of this article will be made available by the authors, without undue reservation.
